# Tachycardiomyopathy a Rare Manifestation of Left Ventricular Outflow Tract Tachycardia. Treatment with Radiofrequency Catheter Ablation

**DOI:** 10.1016/s0972-6292(16)30587-3

**Published:** 2013-01-01

**Authors:** Guillermo Mora, Nohra Romero, van Rendon

**Affiliations:** 1 Associate Professor Department of Internal Medicine, Universidad Nacional de Colombia; 2 Cardiology Service Fundacion Santafe de Bogota

**Keywords:** Idiopathic ventricular tachycardia, left ventricular outflow tract, tachycardiomyopathy

## Introduction

It is recognized that a type of idiopathic ventricular tachycardia (VT) arises from the left ventricular outflow tract (LVOT). This VT exhibits sustained or nonsustained forms, but also appears as frequent premature ventricular contractions (PVCs) of monomorphic QRS morphology [1]. The prognosis is almost uniformly benign. We describe a patient with tachycardiomyopathy resulting from LVOT VT.

## Case Report

**Figure 1:**
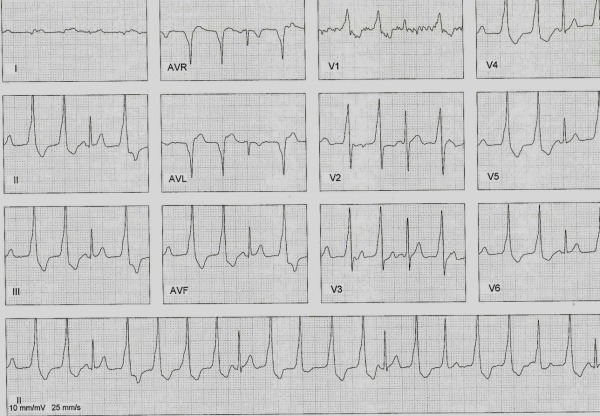
Electrocardiogram showing VT with atrioventricular dissociation and capture beats

An 18 year old man with an incessant monomorphic VT was referred to the electrophysiology service for arrhythmia associated with decreased left ventricular ejection fraction (LVEF). The patient complained of occasional palpitations and was in functional class I. The past history was not significant and physical examination was unremarkable. The electrocardiogram showed wide QRS tachycardia suggestive of VT with a morphology of right bundle branch block (RBBB) and inferior axis (Figure 1). The echocardiogram showed mild left ventricular dilatation and LVEF of 35%. After a written, informed consent was obtained, an electrophysiology study was performed in the postabsortive state and showed incessant monomorphic VT.

**Figure 2:**
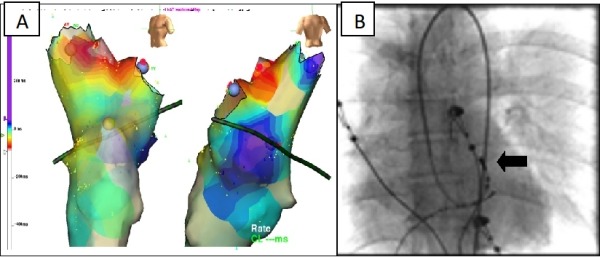
Activation mapping (3-D mapping system Ensite NavX), the earliest activation in the LVOT was found in aortomitral continuity (A). Fluoroscopic image in anteroposterior projection (B), the arrow shows the ablation catheter tip.

**Figure 3:**
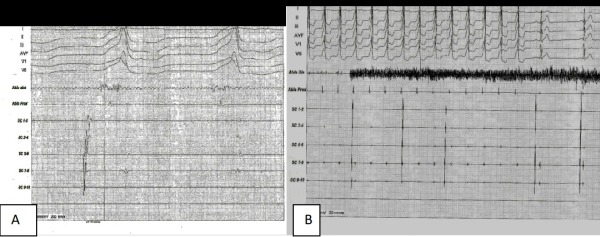
Ablation site electrograms (A) showing prematurity of 17 milliseconds and termination of VT (B) to 5 seconds of the beginning of the application of radiofrequency

**Figure 4:**
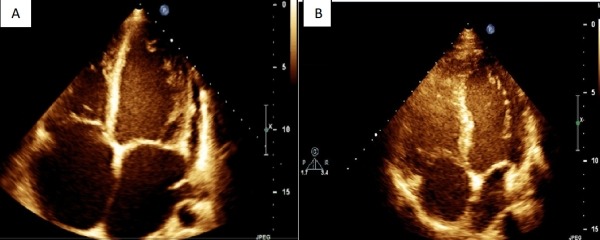
Echocardiographic image apical four-chamber of left ventricle in end-diastole before (A) and after (B) of ablation showing decreasing volumes.

The mapping and ablation were guided by 3-D mapping system (Ensite NavX) with activation mapping. The VT could not be entrained (suggesting a non-reentrant mechanism). The earliest activation in the LVOT was found in aortomitral continuity (Figure 2). One application of radiofrequency resulted in disappearance of VT. (Figure 3). Reinduction of VT at baseline and under the effect of isoproterenol did not produce arrhythmias. After one month, the patient was free from any symptoms and the echocardiogram showed reversal of left ventricular dilatation with LVEF 70% (Figure 4)

## Discussion

The LVOT-VT mechanism is incompletely characterized and probably varies among individual patients. Interestingly, right ventricular outflow tract (RVOT) and LVOT appear to share the same arrhythmogenic mechanism mediated by triggered activity dependent on delayed after- depolarizations.

These arrhythmias present at a relatively early age; VT originating in the RVOT showed a clear predilection in females, whereas those from the LVOT were predominantly seen in males, although this observation has not been confirmed in other studies [1]. The symptoms are palpitations, lightheadedness or presyncope. Most arrhythmias are usually nonsustained, but in some patients additional episodes of sustained VT and in others only sustained VT have been observed. VT can originate from the aortic sinuses, the anterior aspect of the LVOT, the left superior aspect of the interventricular septum, the aorto-mitral continuity, epicardium, and also the superior aspect of the mitral annulus. Crecent fibers of ventricular myocardium have been identified in particular at the base of the left and right aortic sinuses: by contrast the base of the noncoronary cusp is composed of fibrous tissue explaining the very rare incidence of VT originating from this location.

Kumagai et al [2] analyzed 45 consecutive patients with LVOT-VTs; they classified them into VT of aorto-mitral continuity (AMC), anterior site around the mitral annulus (MA), aortic sinus cusps (ASC), and epicardium. Major findings were all the patients with AMC-VT had monophasic R waves in almost all the precordial leads; while those with anterior MA-VT had a morphology with an S wave in many of the precordial leads other than lead V6. The transitional zone was located in lead V1 in the AMC-VT and leads V1-V2 in the anterior MA-VT, while those with ASC-VT has a variable transitional zone in leads V1-4. There were no S waves in lead V6 in any of the groups. The intrinsicoid deflection time in the patients with AMC-VT and anterior MA-VT was significantly greater than that in those with ASC-VT. Finally, there was no significant difference in the R-wave amplitude in the inferior leads in any of the groups. However, AMC-VT and MA-VT groups had small number of patients. In a study by Yomada et al [3] in patients with idiopathic VT, a qrS pattern in leads V1-V3 suggested a site of origin at the junction of the left and right coronary sinuses of Valsalva in the aorta. Recently Chen et al present 10 patients with VT and/or premature ventricular contractions, who had been successfully treated by catheter ablation at the AMC. In this study all patients with anterior AMC location had left bundle brunch block like QRS pattern, in contrast to those with mid-AMC location who had a right bundle brunch block like pattern; R or Rs pattern were observed in the lead V1 in 3 patients y qR pattern in 2 patients. In the other patients were diverse patterns [4]. The R-wave ratio in leads II and III was > 1 in all VTs arising from the AMC [5]. AMC-TV has also been observed in patients with structural heart disease (ischemic, dilated and valvular cardiomyopathy) [5] and appear to be more difficult to ablate and cryosurgery and transcoronary alcohol ablation appear feasible in some cases. Our patient had VT with RBBB morphology, transition in lead V1 and an inferiorly directed axis in the limb leads (Figure 1).

Due to the limited efficacy and side effects of the chronic antiarrhythmic medical treatment, radiofrequency (RF) catheter ablation has been proved effective and is established in the therapy of left outflow tract VT. In most cases, the ablation attempts were approached from the endocardial site. Other reports suggested an epicardial origin successfully eliminated by the transcoronary sinus approach or from the great cardiac vein or anterior interventricular vein without major complications.

Tachycardia-induced cardiomyopathy or tachycardiomyopathy is defined as secondary ventricular dysfunction due to chronic tachycardia, which is fully or partially recoverable after normalization of heart rate. The clinical manifestations of these cases are mixed. Patients with normal heart can suffer from chronic tachyarrhythmias that are well tolerated and asymptomatic, so they consult for the development of heart failure many years after the initiation of tachycardia. By contrast patients with structural heart disease tend to consult earlier. This makes the relationship between the onset of tachycardia and the development of tachycardiomyopathy variable from weeks to years.

The response of ventricular dysfunction to the control of tachycardia is also highly variable, and it is possibly related to myocardial damage caused by long periods of tachycardia and the presence of previous structural heart disease. The first month after the control of tachycardia, is the peak period of recovery of left ventricular function, but there is still improvement at a slower rate in the next 8 to 12 months. Mean duration of reversibility of tachycardiomyopathy in a previous study was 1-15 months in 10 patients [6]

The diagnosis should be suspected in patients with compromised ventricular function in the context of a ventricular or supraventricular tachycardia. The accurate diagnosis can only be established with the recovery of ventricular function after the control of tachycardia. However, it is possible that in some cases the control of heart rate occurs when myocardial damage is irreversible.

The causes of tachycardiomyopathy can be ventricular or supraventricular arrhythmias. Although commonly constant, it has been calculated that if the arrhythmia lasts for more than 15% of the day it can cause ventricular damage [7]. The most common causes are supraventricular arrhythmias. The origin of tachycardiomyopathy in a ventricular arrhythmia is less common, it has been linked to very frequent premature ventricular contractions, ventricular tachycardia of outflow tract of right ventricle, fascicular tachycardia and bundle-branch reentrant tachycardia. Several cases of tachycardiomyopathy treated by RF ablation of VT are published aimed specifically at RVOT VT. Tachycardiomyopathy publications related to tachycardia originating in the left ventricle are less common and correspond to fascicular tachycardia.

Our patient presents a typical case of tachycardiomyopathy secondary to idiopathic LVOT-VT with full recovery of ventricular function after ablation of the tachycardia focus. This is, to our knowledge, the first description of tachycardiomyopathy induced by LOVT-VT and demonstrates the utility of ablation in the cure of this disease.
